# Black Phosphorus, an Emerging Versatile Nanoplatform for Cancer Immunotherapy

**DOI:** 10.3390/pharmaceutics13091344

**Published:** 2021-08-27

**Authors:** Hao Liu, Yijun Mei, Qingqing Zhao, Aining Zhang, Lu Tang, Hongbin Gao, Wei Wang

**Affiliations:** 1 Department of Pharmacy, Guangdong Food and Drug Vocational College, Guangzhou 510520, China; liuh@gdyzy.edu.cn; 2 State Key Laboratory of Natural Medicines, Department of Pharmaceutics, School of Pharmacy, China Pharmaceutical University, Nanjing 210009, China; yjmei@stu.cpu.edu.cn (Y.M.); zhaoquincy@stu.cpu.edu.cn (Q.Z.); zaining@stu.cpu.edu.cn (A.Z.); lutang@stu.cpu.edu.cn (L.T.); 3 NMPA Key Laboratory for Research and Evaluation of Pharmaceutical Preparations and Excipients, China Pharmaceutical University, Nanjing 210009, China; 4 Department of Pharmacy, Baoshan Branch, Renji Hospital, School of Medicine, Shanghai Jiao Tong University, Shanghai 200444, China

**Keywords:** black phosphorus nanomaterial, synergistic therapeutic modality, cancer immunotherapy, photothermal therapy, photodynamic therapy, immune stimulation

## Abstract

Black phosphorus (BP) is one of the emerging versatile nanomaterials with outstanding biocompatibility and biodegradability, exhibiting great potential as a promising inorganic nanomaterial in the biomedical field. BP nanomaterials possess excellent ability for valid bio-conjugation and molecular loading in anticancer therapy. Generally, BP nanomaterials can be classified into BP nanosheets (BPNSs) and BP quantum dots (BPQDs), both of which can be synthesized through various preparation routes. In addition, BP nanomaterials can be applied as photothermal agents (PTA) for the photothermal therapy (PTT) due to their high photothermal conversion efficiency and larger extinction coefficients. The generated local hyperpyrexia leads to thermal elimination of tumor. Besides, BP nanomaterials are capable of producing singlet oxygen, which enable its application as a photosensitizer for photodynamic therapy (PDT). Moreover, BP nanomaterials can be oxidized and degraded to nontoxic phosphonates and phosphate under physiological conditions, improving their safety as a nano drug carrier in cancer therapy. Recently, it has been reported that BP-based PTT is capable of activating immune responses and alleviating the immunosuppressive tumor microenvironment by detection of T lymphocytes and various immunocytokines, indicating that BP-based nanocomposites not only serve as effective PTAs to ablate large solid tumors but also function as an immunomodulation agent to eliminate discrete tumorlets. Therefore, BP-mediated immunotherapy would provide more possibilities for synergistic cancer treatment.

## 1. Introduction

Cancer is a deadly disease that severely threatens public health. Despite the rapid development of modern medicine, effective treatment methods toward cancer are still insufficient. Lack of stability, unsatisfactory therapeutic outcome, unavoidable side effects, and potential drug resistance are typically the main reasons that lead to the failure of anticancer therapy [1]. Nowadays, more and more attempts based on precise drug delivery have been made to ameliorate cancer treatment efficacy. Nanomaterial-based platforms have been widely studied in drug delivery due to their unique properties. They are effective carriers of multiple therapeutic and diagnostic agents due to plenty of advantages, such as localized drug delivery ability, enhanced bioavailability, reduced systemic toxicity, and improved pharmacokinetics through their tunable physicochemical characteristics [2,3]. In addition, nanomaterial-based platforms have captured the researchers’ attention worldwide in the field of both immunotherapy and phototherapy during cancer therapy. For instance, nanomaterials that incorporate immunomodulatory agents such as peptides, nucleic acids, immune checkpoint inhibitors, and other small immunostimulating agents can activate immune cells and modulate the tumor microenvironment to enhance anticancer immunity [4]. Furthermore, nanomaterials also protect sensitive antigens or proteins from degradation or inactivation in the complex physiological environment during cancer immunotherapy. However, insufficient biocompatibility, low biodegradability, and inadequate drug loading ability are the major limitations for the broad application of these nanocarriers [5,6,7]. Therefore, it is essential to adopt a suitable nanoplatform with better anticancer efficacy and fewer aforementioned limitations.

**Figure 1:**
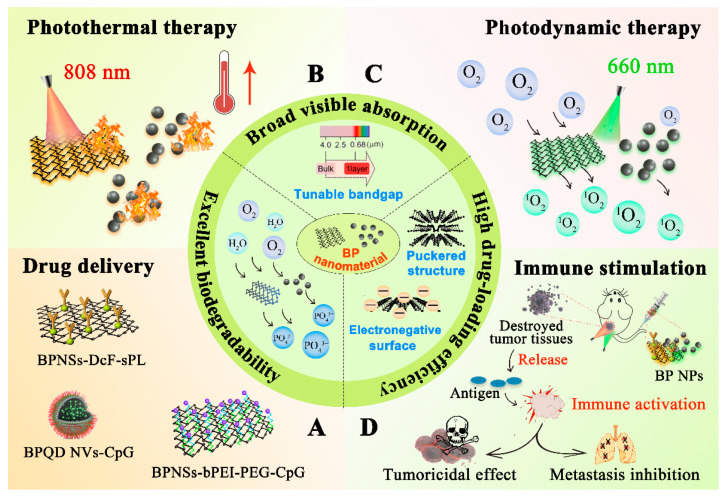
Schematic illustration of the outstanding properties of BP nanomaterials and their applications in anticancer therapy. (**A**) BP nanomaterials are ideal nano drug carriers with excellent agent-loading ability in drug delivery due to their puckered structure, electronegative surface, and excellent biodegradability. (**B**) BP nanomaterials exhibit an outstanding photothermal effect under 808 nm laser irradiation due to their broad visible absorption, leading to hyperthermia and the subsequent tumoricidal effect. (**C**) Due to the unique electronic structure, BP nanomaterials show excellent photodynamic effect under 660 nm laser irradiation, producing large amount of singlet oxygen in local tumor, and eventually leading to tumor destruction. (**D**) Both PTT and PDT can induce immunogenic cell death (ICD) when irradiating the tumor in situ at specific wavelength. Antigens are released in large quantities from the destroyed tumor tissue, which can strongly activate tumor immunity for further producing tumoricidal effect and inhibiting tumor metastasis.

From all the nanomaterials, black phosphorus (BP) stands out as an excellent candidate in the biomedical field. BP is an allotrope of phosphorus which can be prepared through the conversion of white phosphorus (WP) or red phosphorus (RP) under high pressure and high temperature [8]. Nano-scaled BP was firstly synthesized by Li et al. in 2014 [9]. Recently, nano-scaled BP has been obtained through various types of synthesis routes including ultrasonic-assisted liquid-phase exfoliation, wet ball milling, and the solvothermal method. Due to the numerous advantages possessed by BP, it is widely applied in various field such as nanomedicine, detection and sensing, photocatalysts, and optical modulation [10]. Currently, the application of BP as nano drug carriers in anticancer therapy has attracted many researchers’ attention. According to Figure 1, with the property of broad visible absorption band and unique electronic structure, nano-scaled BP exhibits outstanding photothermal conversion efficiency and photosensitivity, which enables it to produce an excellent photothermal and photodynamic effect during cancer therapy, subsequently leading to the devastation of tumor tissue [11]. Moreover, the puckered structure of nano-scaled BP endows it with a huge specific surface area, offering it with a high drug loading ability [12]. In addition, BP can be degraded into nontoxic phosphonates and phosphate after the reaction with water and oxygen in vivo, and coincidentally, phosphorus is an important element that exists abundantly in the human body for osteogenesis, which broadens its application as a kind of biodegradable nanomaterial within the body [13]. From all these mentioned above, BP can be regarded as a safe and multifunctional nanoplatform for anticancer drug delivery [14,15].

However, there are still some obstacles that impede the wide application of BP nanomaterials. For instance, due to its easy reaction with oxygen or water in ambient conditions, the stability of BP is not so good [16]. Surface modification is a suitable solution to prevent BP nanomaterials from being degraded. After the surface functionalization through physical or chemical modification, oxygen or water can be effectively separated from BP nanomaterials, which prevents them from degradation and ensures their original physiochemical properties [17]. Moreover, surface functionalization also endows BP nanomaterials with other properties, such as stronger drug-binding ability, precise tumor-targeting ability, long in vivo circulation time, and enhanced dispersity under physiological conditions. Besides, as an electronegative nano drug carrier with high drug-loading ability, numerous types of positive-charged agents can be loaded onto the surface of BP nanomaterials through electrostatic interaction [18]. The loading of chemotherapeutic drugs and immunostimulatory agents through BP nanomaterials can guarantee the stability of them with a simultaneously reduced administered dosage and corresponding potential side effects.

Currently, the dominated treatment modalities to cancer in clinic are surgery, chemotherapy, and radiotherapy. These cancer therapies aim to eliminate cancer cells but do so at the same expense of normal cells, which sometimes causes serious side effects, accelerating the cancer development in turn. Therefore, a number of newly developed therapeutic strategies including targeted therapy, immunotherapy, gene therapy, and phototherapy have been selectively adopted or in clinical trials [19]. In particular, cancer immunotherapy, which can either instigate or enhance the host immune response to track and destroy cancer cells, has gained considerable attention. Recently, many immunotherapy approaches including monoclonal antibody (mAb) therapy, cytokine therapy, tumor vaccination, checkpoint blockade, and chimeric antigen receptor (CAR) T-cell therapy have been used in clinical studies to treat cancer metastasis [20]. However, many cancers are inherently immunosuppressive and are difficult to control through current immunotherapy [21]. Hence, the combination of topical therapy, such as phototherapy, with immunotherapy can cleverly overcome these limitations as well as amplify the merits of the therapeutic efficacy, which could not only maintain the advantages of immunological approaches, such as systemic immune activation and long-term anticancer immunity, but also increase the selectivity of the overall treatment [22].

Phototherapy, including photothermal therapy (PTT) and photodynamic therapy (PDT), is a non-invasive or slightly invasive therapeutic approach that utilizes laser to deliver energy to the target tissue, providing an alternative solution for primary tumor ablation due to its high specificity in light delivery, low degree of trauma, and effectiveness in destroying the target tumor [23,24]. Near-infrared (NIR) light has been widely applied as the light source for phototherapy due to its deep penetration ability into biological tissues. NIR light can be applied in phototherapy through in situ administration with natural absorbance agents such as photoactive agents or photosensitizers [25]. These photoagents can convert absorbed photon energy into thermal energy, which is termed as PTT for the hyperthermia treatment of cancers, or absorb the specific light energy to generate cytotoxic reactive oxygen species (ROS), which is known as PDT [26]. Therefore, to achieve the goal of eradicating primary tumors and relieving metastasis, a strategy called photoimmunotherapy (PIT) that combines phototherapy and immunotherapy has emerged [27]. PIT is a synergistic treatment approach of which the advantages of both phototherapy and immunotherapy will be augmented while the inherent shortcomings are minimized. As is shown Figure 1D, this combined therapy modality can not only eliminate the primary tumor and clear up residual tumor cells, but also track the metastatic or distant sites, providing more possibilities for patients with advanced tumors.

In the recent five years, biomedical applications of BP have been attached with more and more importance from researchers, and there have been a certain number of reviews that summarized the aforementioned applications from different perspectives. For example, Xiong et al. reviewed BP’s structure, properties, preparation approaches, and techniques to improve BP’s stability and biocompatibility for its further application in the physiological environment [28]. Choi et al. summarized the unique properties of BP through introducing its usage in biosensing, drug delivery, photoacoustic imaging and cancer treatment based on phototherapy [29]. Gui et al. systemically discussed various synthetic methods, physicochemical properties, functionalization, and potential biomedical applications of BP quantum dots (BPQDs) [30]. However, the review on BP’s application in immunotherapy is rare. As BP is an outstanding candidate with excellent optical properties and drug loading capability, this review mainly focuses on the application of BP nanomaterials as drug carriers to load therapeutic agents in cancer immunotherapy; meanwhile, the multifunctionality of BP besides drug carrier in cancer PIT is also highlighted. Moreover, in order to give a comprehensive introduction of this novel material, the other physiochemical properties, synthesis methods, and surface modifications of BP will be outlined as well.

## 2. Properties of Black Phosphorus Nanomaterials

BP nanomaterials have attracted increasing attention in the biomedical field due to their excellent physiochemical properties. For example, BP nanomaterials exhibit great optical characteristics with adjustable bandgap and strong optical absorption, which enable their applications as photothermal agents (PTA) or photosensitizers (PS) for tumor PTT or PDT [31]. Besides, the outstanding biodegradability and biocompatibility of BP nanomaterials render their possible applications as biomaterials with fewer side effects in cancer therapy [32]. Moreover, due to the unique surface structure, BP nanomaterials possess high drug-loading efficiency, enabling their utilization as drug carriers. Thus, BP nanomaterials are regarded as a superior candidate in nano-based anticancer therapy.

### 2.1. Optical Properties

BP possesses a highly thickness-dependent direct bandgap, varying from 0.3 eV for bulk BP to 2.0 eV for nano-scaled BP [33]. To BP nanosheets (BPNSs), the optical bandgap can be finely tuned by regulating the number of layers, which allows BPNSs to absorb a broad range of wavelengths. Additionally, BP nanomaterials exhibit a large NIR extinction coefficient and high photothermal conversion efficiency. As a result, BP nanomaterials can serve as effective PTA for PTT [34]. Therefore, the energy of incident light can be converted to heat through BP nanomaterials, thereby leading to photothermal ablation of tumors under NIR irradiation [35]. Besides, upon illumination, the energy from the excited excitons of BP nanomaterials can be transferred to the surrounding O_2_, which subsequently leads to the change of O_2_’s electronic configuration [36]. Furthermore, eventually, singlet oxygen (^1^O_2_) can be generated at a high level, which is the key factor for PDT-induced tumor cell apoptosis [37].

### 2.2. Biocompatibility and Biodegradability

As one of the most common bio-components existing in human body, phosphorus makes up approximately 1% of the overall body weight and this important element is closely connected to the formation of cell membranes and deoxyribonucleic acid (DNA), as well as the mineral components of hard tissue [38]. The lone-pair electrons on each phosphorus atom cause the relatively high reactivity of BP toward water and oxygen, leading to the subsequent degradation of BP into nontoxic phosphates (PO_4_^3−^) and other PxOy species in the physiological environment [39,40]. The PO_4_^3−^ traps the surrounding positive calcium ions (Ca^2+^) to form calcium phosphate (CaP), which can facilitate local biomineralization for in situ bone regeneration [41]. Although analogs of PO_4_^3−^ are the common form of existence for molecules containing phosphorus in our body, transient elevation of cytosolic PO_4_^3−^ can affect oxidative stress, inhibit proliferation of cancer cells, and eventually induce apoptosis [42].

### 2.3. High Loading Efficiency

As a novel drug delivery carrier, BP nanomaterials exhibit high drug-loading capacity because of the corrugated crystalline and textural properties [43]. Nano-scaled BP possesses a puckered structure, endowing it with a large surface area-to-volume ratio to load therapeutic drugs with high efficiency [44]. For example, Zhou et al. constructed a BPNSs-based complex loading CRISPR-associated protein 9 (Cas9) ribonucleoprotein with high loading efficiency, which showed a remarkable Cas9 ribonucleoprotein loading capacity of 98.7% on BPNSs [45]. In addition, doxorubicin (DOX) was absorbed onto BPNSs via electrostatic interaction by Chen et al., and the drug-loaded nanosheets even showed 950% loading capacity for DOX in weight, which was higher than any previously reported nanomaterials [23].

## 3. Synthesis of Black Phosphorus Nanomaterials

BP nanomaterials can be divided into BPNSs and BPQDs based on their spatial dimensions. BPNSs belong to two-dimensional materials, while BPQDs exhibit a zero-dimensional characteristic. As is shown in Table 1, the preparation methods of BPNSs can be mainly classified into mechanical exfoliation, ultrasonic-assisted liquid-phase exfoliation, electrochemical exfoliation, and chemical vapor deposition, while ultrasonic exfoliation, solvothermal method, and pulsed laser ablation are three important routes for the preparation of BPQDs.

### 3.1. Synthesis of Black Phosphorus Nanosheets

As a novel inorganic nanomaterial with numerous characteristics which can be applied in various fields, the role of BPNSs was explored by more and more researchers. Meanwhile, in order to exert the effectiveness of BPNSs in certain fields, it is essential to seek a proper way for the synthesis of BPNSs. The electronic, optical, and thermal properties of BPNSs can be affected in some extent related to the eventual size of the obtained BPNSs. The main preparation technologies of BPNSs are respectively introduced in the following content.

#### 3.1.1. Mechanical Exfoliation Method

Owing to the weak van der Waals bond existing among the interlayers of BPNSs, various layers of BP flakes can be obtained through mechanical exfoliation method [62]. Tape exfoliation and wet ball milling (WBM) exfoliation are two common mechanical exfoliation methods for the synthesis of BPNSs.

**Figure 2:**
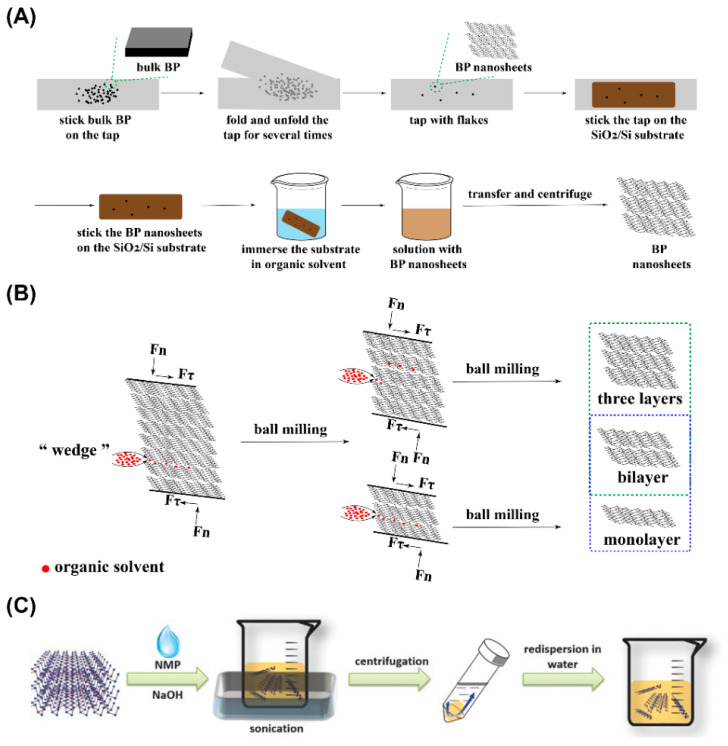
Synthesis routes of BPNSs based on (**A**) tape exfoliation method, (**B**) wet ball milling exfoliation method, and (**C**) ultrasonic-assisted liquid-phase exfoliation method (Reproduced with permission from [48], WILEY, 2015).

*Tape Exfoliation Method* Tape exfoliation is a relatively traditional method. Tape, substrate (usually SiO_2_/Si), and bulk BP are three necessities if BPNSs are synthesized through this method. As is showed in Figure 2A, the tape exfoliation method can be generally divided into the following steps: (1) the precursor material (usually a bulk BP) is placed on the tape; (2) the tape is folded and unfolded about 10 times; (3) the flakes are scattered on the tape; (4) the tape containing flakes is transferred to the substrate (usually SiO_2_/Si); (5) the substrate with flakes attached is placed in an organic solvent (usually acetone) to obtain free flakes [63,64].

However, the thickness of the BPNSs prepared through this method is usually uneven. Additionally, the lateral size of the obtained BPNSs is small. In order to circumvent the inadequate lateral dimensions of BPNSs, Guan et al. prepared an Au/Ag deposited SiO_2_/Si substrate to expand the adhesion between BP and the substrate [46]. The lateral size of the multi-layer BP obtained in this research was as wide as 50 μm, and the cross-sectional area was 100 times that of the conventional tape exfoliation method.

*Wet Ball Milling Exfoliation Method* As is exhibited in Figure 2B, the solvent can be infiltrated into bulk BP by grinding force through the WBM exfoliation method. Furthermore, the weak interlayer van der Waals force of bulk BP will be destroyed to obtain BPNSs with different layers. Fewer types of organic solvent are needed during WBM process, and the solid/liquid ratio was 100:1 (mg/mL) for WBM exfoliation [47]. Liu et al. compared various kinds of organic solvents (including dimethyl sulfoxide (DMSO), N-methyl pyrrolidone (NMP), N, N-dimethylformamide (DMF), ethylene glycol (EG), and absolute ethanol (AE) on the exfoliation effect through WBM exfoliation method [47]. The results showed that DMSO was the best organic solvent for WBM exfoliation, enabling 3~5 layers of BPNSs to be obtained.

#### 3.1.2. Ultrasonic-Assisted Liquid-Phase Exfoliation Method

According to Figure 2C, ultrasonic-assisted liquid-phase exfoliation can be divided into three steps: (1) solvent addition to the bulk BP; (2) ultrasound adoption for BP exfoliation; (3) the purification and collection of BPNSs [65]. In this method, the ultrasonic power, ultrasonic time, and the type of selected organic solvent will affect the quality of prepared BPNSs. Due to the high oxidizability of BP in ambient condition, the applied organic solvent needs to be carefully deoxidized before exfoliation. Isopropanol (IPA), DMSO, N-vinylpyrrolidone (NVP), N-cyclohexyl-2-pyrrolidone (CHP), 1,2-dichlorobenzene (DCB), DMF, acetone, ethanol, and methanol are all available to be applied as the solvent during ultrasonic-assisted liquid-phase exfoliation. Su et al. studied the exfoliation efficiency of BP in different solvents (IPA, DMSO, VNP, CHP, DCB, DMF, acetone, ethanol, and methanol) assisted by Li_2_SiF_6_ [66]. The results showed that the effect on the production yield and concentration of BPNSs in the DMSO group were the best. The exfoliation efficiency in the DMSO group could reach 75%. The study also showed that the higher the surface tension of the solvent, the higher the yield and concentration of BPNSs. As a multifunctional organic polymer, polyvinylpyrrolidone (PVP) can be applied to improve the water dispersibility of hydrophobic materials. The present of PVP can influence the exfoliation of solvents. Shen et al. compared the exfoliation efficiency of BP in different solvents (including IPA, EtOH, NMP, and DMF) with presence of PVP, and the results showed that PVP significantly enhanced the stability of BPNSs [67].

#### 3.1.3. Electrochemical Exfoliation Method

Electrochemical exfoliation is a green, environmentally friendly, highly controllable, and simple exfoliation method. Electrochemical exfoliation equipment includes an anode and a cathode. The exfoliation effect can be eventually achieved by placing bulk BP at different electrode positions, replenishing electrolyte, and applying a certain voltage. The electrolyte commonly used in anodic exfoliation is sulfuric acid (H_2_SO_4_) or sulfate solution (SO_4_^2−^), and the solvent is H_2_O. Tetraheptylammonium bromide (THAB), Tetrabutylammonium hexafluorophosphate (TBAPF6), Tetraalkylammonium tetrafluoroborate (TAA), Tetraethylammonium perchlorate (TEAP), and Tetrabutylphosphonium bromide (TBPB) can be applied as the electrolyte of cathodic exfoliation, and the solvent could be DMF, H_2_O, Propylene carbonate (PC), and DMSO [68].

Ambrosi et al. applied BP crystals that were prepared from red phosphorus through thermal conversion as the anode, platinum (Pt) foil as the cathode, and 0.5 M H_2_SO_4_ as the electrolyte [51]. First, a voltage of 1 V was applied for 2 min to promote wetting, and then the voltage was adjusted to 3 V for 2 h to exfoliate BP crystals. This method could endow BPNSs with a single-layer or multi-layer structure. The experimental results showed that this method possessed a certain potential to reduce the lateral dimension and the thickness of BPNSs, and BP powder could be placed at the cathode to obtain BPNSs with the thickness of 2–7 nm [50].

#### 3.1.4. Chemical Vapor Deposition Method

Chemical vapor deposition (CVD) is another common method to prepare BPNSs. One or several precursor substances are necessary to generate a thin layer of products on the gas phase or solid substrate. This method usually requires certain external stimuli, such as heat, high energy radiation, or plasma. In such an environment, the precursor substance will undergo a change in chemical structure and then be transformed into the desired product. Smith et al. used amorphous thin film of red phosphorus to generate BPNSs in situ on the surface of silicon substrate [53]. The surface area of the BPNSs obtained by this synthesis method could reach more than 3 μm^2^. The study also pointed out that a large area of multi-layer BP can be obtained for practical application under proper temperature or pressure condition.

In general, the application of BPNSs becomes more and more widespread. Furthermore, a variety of methods to synthesize BPNSs have also been developed to meet certain application requirements. In addition, many other preparation methods are also reported or used for the synthesis of BPNSs, such as laser exfoliation, gas-phase growth strategy, plus-laser deposition, and the solvothermal method [54,55,69,70].

### 3.2. Synthesis of Black Phosphorus Quantum Dots

BPQDs are another form of BP nanomaterials that belong to zero-dimensional nanomaterial. The applications of BPQDs in tumor phototherapy, electronic sensing, and bio-imaging have attracted great attention after Zhang et al. synthesized BPQDs for the first time [59,71,72]. Nowadays, more and more preparation methods such as ultrasonic exfoliation, the solvothermal method, and pulsed laser ablation have been developed to fabricate BPQDs.

#### 3.2.1. Ultrasonic Exfoliation Method

**Figure 3:**
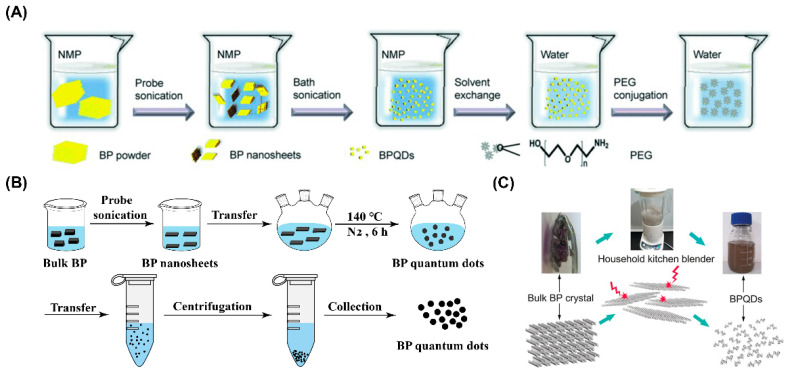
Synthesis routes of BPQDs based on (**A**) ultrasonic exfoliation method (Reproduced with permission from [57], WILEY, 2015), (**B**) the solvothermal method, and (**C**) blender breaking method (Reproduced with permission from [61], WILEY, 2016).

Ultrasonic exfoliation is a common route for the preparation of BPQDs. As is described in Figure 3A, NMP is often applied as the solvent for ultrasonic exfoliation to synthesize BPQDs [71]. Additionally, other solvents, such as deionized water and IPA, are also available to prepare BPQDs through this method [73,74]. The power of ultrasonic exfoliation, the ultrasonic system mode, and reaction temperature can be adjusted according to the experimental requirement [57,75].

Li et al. applied liquid phase exfoliation method to prepare ultra-small BPQDs from milled BP crystals powder [31]. Dual-power ultrasound was performed in this synthesis. First, the mixture solution of milled BP crystals powder and NMP was sonicated in a 600 W ultrasonic cell disruption system for 6 h, and then they were sonicated with a 300 W ultrasonic cleaning system for another 10 h to prepare the BPQDs suspension. Finally, the obtained suspension was purified by centrifugation to collect pure BPQDs. Transmission electron microscope (TEM) images and atomic force microscope (AFM) images showed that the particle size of single dispersed BPQDs was 2.5 ± 0.7 nm, and the average height of BPQDs was 1.3 ± 0.7 nm, respectively, which was close to 1–2 layers of BP.

#### 3.2.2. Solvothermal Method

The solvothermal method is another method for the preparation of BPQDs. According to Figure 3B, NMP was applied as the solvent and BP powder as the raw material to prepare BPQDs under nitrogen atmosphere at 140 °C [76]. To the raw material bulk BP crystals, ultrasonic pretreatment is performed before the solvothermal method [77]. Wang et al. used a two-step method to synthesize BPQDs [58]. Firstly, the BP bulk crystals stored in the Argon (Ar) glovebox was sonicated by probe to fabricate BP powder. Subsequently, the obtained BP powder, NaOH, and NMP solution were mixed in a bottle, and the entire system was heated and stirred for 6 h at 140 °C under N_2_ environment. Finally, the mixed suspension was centrifuged at 7000 rpm for 20 min to collect the supernatant containing BPQDs. The results of TEM and AFM exhibited that the prepared BPQDs possessed the average particle size of 2.1 ± 0.1 nm, and the typical height of 2.0 ± 1.0 nm, respectively.

#### 3.2.3. Blender Breaking Method

Blender breaking is a novel method for the synthesis of BPQDs. As is exhibited in Figure 3C, complicated equipment is not required when preparing BPQDs through the blender breaking method. Instead, only a household kitchen blender is necessary for blender breaking, which can crush bulk BP into BPQDs with ultra-small particle size under the powerful crushing force of blender. Therefore, the blender breaking method can be regarded as a convenient and rapid method for the synthesis of BPQDs. Zhu et al. adopted bulk BP crystals as raw materials and DMSO as the solvent to prepare BPQDs through a household kitchen blender [61]. Under the effect of high turbulent shear rate, the layer-to-layer disintegration of bulk BP crystals was achieved to form BPQDs. TEM images showed that the average particle size of BPQDs prepared by this method was 2.2 ± 0.4 nm. The photothermal properties of the BPQDs dispersion obtained in this study indicated that the temperature of the BPQDs dispersion increased from 18.3 to 50.1 °C under the irradiation of 808 nm laser for 7 min. In comparison, there was ignorable temperature change in the pure water group with the same treatment, indicating that the prepared BPQDs possessed excellent photothermal properties.

#### 3.2.4. Pulsed Laser Ablation Method

Compared with ultrasonic exfoliation and solvothermal methods, the pulsed laser ablation (PLA) method is a time-consuming method to prepare BPQDs. Ren et al. adopted PLA method to prepare BPQDs with an average diameter of 6 nm and a height of 1.1 nm [60]. Moreover, fluorescence quantum yield of the prepared BPQDs was as high as 20.7%. In this method, bulk BP crystals were first placed in a cuvette containing isopropyl ether with N_2_ deoxygenation, and then a polystyrene cover was used to seal the top opening and a sealing film to isolate oxygen. Finally, the entire system was fixed on a three-dimensional console and exposed to a 140 mW/cm^2^ 1064 nm Nd:YAG pulsed laser for 30 min to obtain yellow suspension containing BPQDs. The pulse width of the laser in this experiment was 3–6 ns and a repetition rate was 10 Hz.

According to the aforementioned introduction of the synthesis of BPQDs, it is concluded that the prepared BPQDs possessed the average size less than 10 nm. The small-size property of BPQDs not only enables them to be more biocompatible and biodegradable, but endows BPQDs with more application possibilities, especially in cancer therapy [59].

## 4. Surface Modification of Black Phosphorus Nanomaterials

**Figure 4:**
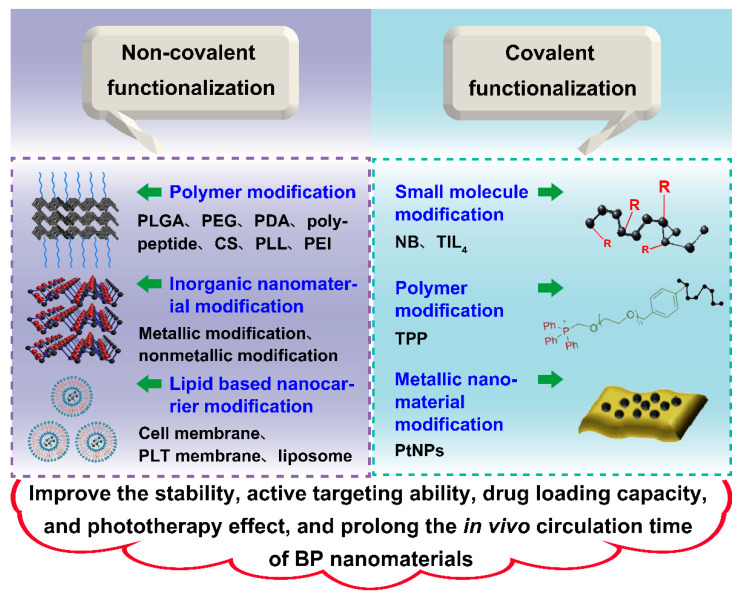
Schematic illustration of the surface modification of BP nanomaterials. BP nanomaterials can be functionalized non-covalently or covalently for property improvement. Non-covalent functionalization can be further classified into polymer modification, inorganic nanomaterial modification, and lipid-based nanocarrier modification. Furthermore, covalent functionalization can be further classified into small molecule modification, polymer modification, and metallic nanomaterial modification.

As is mentioned above, BP nanomaterials are promising materials that can be used in drug delivery system (DDS) in the field of cancer therapy, however, the instability of this nanomaterial under ambient condition limits its broad application to a great extent. The surface modification of BP nanomaterials can be an effective way to circumvent this problem. Moreover, surface modification is also beneficial to improve other properties of BP nanomaterials, such as active targeting ability, prolonged in vivo circulation time, enhanced photothermal or photodynamic capability, and improved drug loading capacity. As is depicted in Figure 4, surface modification includes physical modification and chemical modification, which both play important roles in improving the overall properties of BP nanomaterials.

### 4.1. Physical Modification

Physical modification is a type of surface modification method without chemical reaction. Therefore, the modification process is usually simpler in contrast to chemical modification. In addition, the property of each component in nano DDS can be well preserved due to the mild modification condition during physical modification. As is shown in Table 2, modification using a polymer, inorganic nanomaterials, and a lipid-based nanocarrier are the common physical modification methods for BP nanomaterials during the construction of nano DDS.

#### 4.1.1. Modification Using Polymers

Organic polymers, such as poly (lactic-co-glycolic acid) (PLGA), polyethylene glycol (PEG), polydopamine (PDA), and polypeptide, can coat on the surface of BP nanomaterials, and subsequently improve their properties.

**PLGA Modification**: PLGA, which has been approved for use in clinic by the Food and Drug Administration (FDA), is a biocompatible organic polymer obtained by the polymerization of lactic acid and glycolic acid [98,99]. PLGA can be eventually degraded into nontoxic substances in vivo. Thus, the biocompatibility and biodegradability of BP nanomaterials after the surface modification of PLGA can be fully ensured. Wang et al. loaded BPQDs into PLGA with the additional conjugation of a chemotherapeutic agent docetaxel (DTX) to form a nano DDS named BP/DTX@PLGA [79]. After intravenous (i.v.) administration, this nano DDS exhibited excellent biocompatibility and outstanding controllable chemophotothermal combinatory therapeutic effect during anticancer treatment. Furthermore, PLGA modification not only endowed the nano DDS with outstanding monodispersity in solution, but let the nano DDS possess a proper particle size for tumor targeting through enhanced permeability and retention (EPR) effect as well.

Compared with original PLGA, structure modified PLGA can better improve the properties of nano DDS containing nano-scaled BP. Chan et al. synthesized PLGA_-ss-_D through the covalent modification of PLGA with cystamine and oxalic acid modified polyethylenimine (PEI), targeting polypeptide peptide motif Arg-Gly-Asp-Gys (RGD), and 2,3-dimethylmaleic anhydride (DMMA) [81]. Cystamine with disulfide linkage can be regarded as the bioresponsive trigger that can be broken by the reductive glutathione (GSH) which is rich in the tumor microenvironment (TME), while DMMA endows the nano DDS with surface charge-switching property [100,101]. After modifying BPQDs with PLGA_-ss-_D, this nano DDS showed surface-charge-switching ability, pH-responsive property, cancer-targeting ability, accurate tumor-specific release, prolonged blood circulation, and precise tumor radiosensitization during anticancer therapy.

**PEG Modification**: PEG is usually polymerized through glycol or the reaction between ethylene oxide and water. PEG can be regarded as an ideal surface modification polymer during nano DDS preparation, mainly due to its nonimmunogenicity, nonantigenicity, and protein rejection [102]. Wan et al. modified BPNSs with PEG through electrostatic interactions, resulting in the enhancement of biocompatibility and physiological stability of BPNSs [82]. Additionally, the photothermal conversion effect of BPNSs was ensured after PEGylation. PEGylated BPNSs exhibited a synergistic effect with imiquimod (R837) applied after PTT in immunostimulation and tumor immunotherapy.

Modified PEG can show more unique characteristics compared with original PEG. Wu et al. functionalized PEG with dibenzaldehyde (DF) and polyaspartylhydrazide (PAHy) covalently, forming DF-PEG-PAHy for cancer treatment [83]. After the physical modification of BPNSs through DF-PEG-PAHy and the encapsulation of DOX, the obtained DDS exhibited the microstructure of hydrogel with various novel properties, such as excellent gelation characteristics, pH sensitivity, and near-infrared responsiveness. Besides, the biocompatibility and the photothermal effect of the DDS are satisfying both in vitro and in vivo.

**PDA Modification**: PDA can be prepared through the self-polymerization of dopamine (DA) under alkaline condition [103]. PDA possesses a good photothermal effect, which can endow DDS with photothermal conversion capability, or produce a synergistic effect on photothermal conversion efficiency [104]. Moreover, PDA modification is also beneficial to enhance the stability of DDS. Yang et al. coated BPNSs with PDA, forming a conformal layer on the surface of BPNSs. This modification effectively improved the stability of BPNSs, enhanced the photothermal conversion efficiency, and provided amine anchors for the further covalent functionalization of DDS by chlorin e6 (Ce6) and triphenyl phosphonium (TPP). After i.v. injection of BP@PDA-Ce6 and TPP nanocomposites to tumor-bearing mice, this nano DDS exhibited precise mitochondria-targeting ability and photothermal/photodynamic synergistic effect during cancer therapy.

Poly (2-ethyl-2-oxazoline) (PEOz) is a long-chain polymer approved by FDA. PEOz can be regarded as a suitable substitute to PEG mainly due to its long circulation and ability to circumvent protein adsorption and blood clearance in vivo [105,106]. Gao et al. applied PEOz conjugated PDA for the surface modification of BPNSs. This DDS not only showed improved photothermal conversion efficiency and biostability, but also exhibited enhanced targeted ability, a long circulation property, pH-responsiveness, and a photo-responsive drug release characteristic in vivo. Moreover, this DDS could efficiently load the chemotherapy drug DOX, synergistically exerted anticancer effect through chemotherapy and photothermal therapy.

**Polypeptide Modification**: Polypeptide modification is another surface modification method that does good to the improvement of BP nanomaterials’ stability. Wang et al. synthesized a tailored polypeptide named Fmoc-Lys-Lys-Phe (Fmoc-KKF) [86]. After the surface modification of BPNSs, the Fmoc-KKF covered on the surface of BPNSs subsequently prevented the reaction between BPNSs and oxygen. Eventually, the degradation of BPNSs was effectively retarded. Meanwhile, the cellular uptake of this nano DDS by human cervical cancer cell HeLa was also enhanced.

**Other Modifications**: Except for these surface modification approaches described above, other types of organic polymer, such as polylysine (PLL), chitosan (CS), and PEI, can also be applied to modify BP nanomaterials physically for the characteristic improvement. For example, Yue et al. combined PLL with BP through electrostatic adsorption for improving the cell adhesion, membrane penetration, and stability of the nano DDS [88]. Wang et al. modified BPNSs with CS, acquiring a DDS exhibiting excellent ROS production ability and tumor growth inhibition effect [87]. Zhang et al. modified BPNSs with PEI through electrostatic adsorption [89]. This PEI modification endowed BPNSs with the ability to be further functionalized. Besides, the stability of BPNSs was simultaneously improved after PEI modification.

#### 4.1.2. Modification Using Inorganic Nanomaterials

Inorganic modification is another important type of surface modification method to improve BP nanomaterials’ properties. Inorganic modification can be further classified into two categories: one is modification through metallic nanomaterials and the other is modification through nonmetallic nanomaterials.

**Metallic NanomaterialModification**: Distinct metallic nanomaterials possess different unique characteristics. Thus, these characteristics can be obtained by BP nanomaterials with the surface modification through metallic compound. Aurum (Au) is an important metal element which can endow BP nanomaterials with diverse properties. Yang and his teammates modified BPNSs with Au nanoparticles (AuNPs) through a one-step facile synthetic method [90]. In contrast to bare BPNSs, the obtained BP-Au NSs exhibited enhanced photothermal efficiency. Additionally, the modification through AuNPs enabled BP-Au NSs to act as effective surface-enhanced Raman scattering (SERS) substrates for Raman biodetection. Thus, the synthesized BP-Au NSs could not only destroy cancer cells, but showed outstanding SERS activity to monitor the photothermal effect during cancer treatment by Raman technique as well.

The iridium (Ir) complex is a kind of inorganic compound with satisfying photodynamic property, which shows the potential to produce a synergistic effect with BP nanomaterials in anticancer treatment [107]. Chan et al. modified BPNSs through the application of unsaturated Ir complex to synthesize a two-dimensional layered nanosystem [92]. This modification enhanced the photoelectric characteristics and photo-induced charge carrier dynamics of BPNSs, leading to the more effective generation of single oxygen through the nanosystem after X-ray irradiation. In addition, the combination of Ir complex enabled RGD peptide to further modify the nanosystem, endowing the whole nanosystem with precise targeting ability. The obtained RGD-Ir@BP nanosystem showed a highly efficient and safe property in cancer radiotherapy.

**Nonmetallic Nanomaterial Modification**: Modification through nonmetallic nanomaterial is another form of inorganic modification to BP nanomaterials. Mesoporous silica (MS) is a promising nanomaterial in biomedical field due to its excellent biocompatibility, tunable pore size, and large pore volume. Besides, MS possesses good water dispersion effect and surface functionalization possibility because of its hydrophilic surface silanol with high surface area. Chen et al. functionalized BPNSs through the surface modification through MS via surfactant-assisted co-assembly [91]. The coating of MS around BPNSs not only strengthened BPNSs’ dispersity, improved drug loading capacity, and enhanced photothermal conversion efficiency, but also ensured further surface modification with other targeting material. This nano DDS could exert chemo-photothermal synergistic tumor-targeted therapy effect with effective metastasis inhibition results during cancer treatment.

#### 4.1.3. Modification Using Lipid Based Nanocarrier

A drug carrier plays an importance role in drug delivery and brings various benefits in contrast to the direct application of free drugs, such as enhanced stability, decreased toxicity, improved dispersity, precise targeting effect, and prolonged systemic circulation time. Among numerous drug carriers, cell membrane, platelet membrane (PLTm), and liposome are three types of drug carrier which are widely used in the modification of BP nanomaterials.

**Cell Membrane Modification**: Cell membrane can be subdivided into several categories including red blood cell membrane (RM), neutrophil membrane, tumor cell membrane, and macrophage membrane. Cell membrane is usually obtained from the original cell. Therefore, the nano DDS camouflaged by the cell membrane usually possesses the ability to replicate the highly complex cellular functionalities to create new therapeutic modalities [108]. RM is a multifunctional drug carrier with outstanding biocompatibility. Moreover, the modification through RM can protect NPs from being removed from the body, eventually acquiring a long circulation effect [109,110]. Liang et al. coated BPQDs by RMs, forming a BPQD-RM nanovesicle biomimetic formulation with long circulation time and precise tumor accumulation ability in vivo [93]. After the in situ NIR irradiation with the combination therapy of programmed cell death protein 1 (PD-1) antibody (aPD-1), the growth of both original and metastatic tumor was obviously inhibited.

The tumor cell membrane is generally collected from tumor tissue or tumor cells. NPs modified with tumor cell membrane can be regarded as tumor vaccine, and specific response of the immune system can be enhanced after the administration of tumor cell membrane-cloaked NPs. Ye et al. functionalized BPQDs with surgically removed tumor cell membrane, forming BPQD-CCNVs as a type of photothermal cancer vaccine [94]. With the existence of lipopolysaccharide (LPS), GM-CSF, and aPD-1, a strong and durable immunological response was induced after treatment, subsequently inhibited tumor recurrence and metastasis.

**Platelet Membrane Modification**: Platelet (PLT) possesses the ability to target to tumor tissue through its interaction with the P-selectin and CD44 receptor expressed on tumor cells, and the PLTm extract from PLT inherits these aforementioned properties [111]. Therefore, the camouflage of PLTm endows NPs with the ability to target to tumor cells actively. In addition, modification through PLTm prolongs the retention time of NPs in vivo through circumventing macrophage uptake and the activation of complements in autogenous plasma [95,112]. Shang et al. coated BPQDs with PLTm carrying an anticancer agent named Hederagenin (HED), forming the nano DDS named PLTm@BPQDs-HED [95]. The functionalization of PLTm enables this nano DDS to load chemotherapeutic drug with high efficiency and can target to tumor sites precisely. Subsequently, both the tumor cell viability and the mitochondrial membrane potential (MMP) were obviously reduced, achieving an excellent tumoricidal effect during anticancer treatment.

**Liposome Modification**: Liposome is a traditional drug carrier that has been widely applied in anticancer therapy. Liposome has numerous advantages that are beneficial to the encapsulated agents. For example, the modification of liposome reduces drug toxicity, enhances drug stability, and prolongs drug’s circulation time in vivo [113,114]. Zhang et al. co-encapsulated BPQDs and colon cancer cells derived neoantigen peptide Adpgk into liposome, obtaining Adpgk-BPQDs-liposome as a therapeutic vaccine [97]. This liposome encapsulation effectively prevented the degradation of BPQDs, and further maintained their photothermal characteristics in PBS. After combining this DDS with F127 gel and an immune adjuvants GM-CSF, strong tumor immune response was stimulated, exerting an outstanding anticancer effect through the enhancement of tumor immunity.

In contrast to conventional liposome, a multifunctional liposome (MFL) usually shows unique characteristics depending on various functionalization methods. Hai et al. designed a type of folate (FA)-modified liposome with the encapsulation of resveratrol (RV) and catalase-loaded BPNSs for photothermal drug delivery and oxygen self-enriched photodynamic therapy [96]. The FA existed on the surface of liposome enabled the nano DDS to selectively target to cancer cells, subsequently releasing the encapsulated agents from the nano DDS to exert anticancer effects. Besides, the imaging units conjugated on the surface of liposome NPs ensured precise disease diagnosis during cancer treatment.

### 4.2. Chemical Modification

Although the process of surface functionalization of BP nanomaterials through physical modification is relatively simple, sometimes the bonding strength between BP nanomaterials and modifier may be inadequate, which may lead to unexpected decomposition of DDS. In contrast to physical modification, the modifier can more tightly conjugate onto the surface of BP nanomaterials through chemical modification due to the strong covalent bond formed between the P atom and the modifier [115,116]. Commonly, chemical modification of BP nanomaterials can be divided into three categories, namely modification using small molecule, modification using polymer, and modification using metallic compound. Table 3 demonstrates various examples regarding the chemical modification of BP nanomaterials in detail.

#### 4.2.1. Modification Using Small Molecule

Small molecule is a kind of substance with the molecular weight less than 500 Da [121]. Some types of small molecules, such as Nile blue (NB) and Titanium sulfonate ligand (TIL_4_), can be applied as suitable surface modifier for BP nanomaterials through chemical modification. The covalent conjugation of these small molecules effectively improves BP nanomaterials’ properties when constructing DDS.

**Nile Blue Modification**: Among numerous small molecules, NB is a multifunctional dye with outstanding fluorescence property. After converting NB into its diazonium tetrafluoroborate salt (NB-D), the formation of covalent bond between BP and NB is accessible through aryl diazonium coupling [122]. Zhao et al. covalently modified BPNSs with NB through the synthesis route described above, forming the dye-modified BPNSs named NB@BP [117]. This NP exhibited enhanced storage stability, outstanding photothermal tumor ablation effect, NIR imaging efficiency, and excellent biocompatibility, showing a bright application future in the field of anticancer therapy.

**Titanium Sulfonate Ligand Modification**: TIL_4_ is an important type of small molecule that can physically modify BP nanomaterials’ surface through coordinate covalent bonds. After combining BP nanomaterials with TIL_4_, The P-Ti coordination occupies the lone pair electrons on P atoms, preventing the oxidation of BP nanomaterials in air and water, eventually ensuring both the stability and photothermal conversion effect of BP nanomaterials [123]. Qu et al. modified BPNSs with TIL_4_ synthesized by their research team, forming TiL_4_@BPNSs with reduced cytotoxicity and pro-inflammation [118]. Furthermore, the functionalization of TiL_4_ prevented BPNSs from being uptaken by macrophages, leading to the prolonged circulation in vivo. Meanwhile, TiL_4_@BPNSs exhibited improved biocompatibility due to the modification of TIL_4_. Therefore, TiL_4_@BPNSs also possessed excellent application prospect in anticancer therapy.

#### 4.2.2. Modification Using Polymer

TPP is a small organic molecule with the precise targeting ability to mitochondria. Aryl diazo (AD) group can react with the P atom on BP to form stable covalent bonds. Thus, Qi et al. firstly functionalized PEG with both TPP and AD, endowing PEG with the ability to chemically react with BPQDs and possess precise targeting ability to mitochondria [119]. Subsequently, BPQD-PEG-TPP nano DDS was acquired through the reaction between BPQDs and TPP-PEG-AD. After i.v. administration, this nano DDS exhibited higher affinity to mitochondria of the tumor cell and more ROS production through photothermal effect in contrast to bare BPQDs. In addition, the modification of TPP-PEG-AD effectively improved the stability and dispersibility of the nano DDS under physiological condition with negligible side effect. Therefore, the study of this nano DDS provides a novel idea for anticancer therapy based on mitochondria targeting.

#### 4.2.3. Modification Using Metallic Nanomaterial

Some inorganic substances can also modify BP nanomaterials’ surface through chemical modification. Platinum nanoparticles (PtNPs) are used as the catalyst to decompose H_2_O_2_. Yang et al. anchored catalase-like PtNPs onto the surface of BPNSs through coordinating covalent bonds, endowing the DDS with the ability to eliminate the overexpressed H_2_O_2_ in TME [120]. Oxygen was progressively released during the degradation process of H_2_O_2_, subsequently enhancing the photodynamic effect of the nano DDS during cancer treatment. Moreover, due to the empty orbit exists on PtNPs, it is feasible to conjugate PtNPs to the substance with a lone pair of electrons such as thiol compounds [124]. Therefore, the surface modification with PtNPs may further enhance the modifiability of BP nanocomposites to generate stronger anticancer effect.

## 5. Delivery of Immunotherapeutic Agents for Cancer Immunotherapy

As a versatile inorganic nanoplatform, various researches based on BP nanomaterials have been conducted in biomedical field including cancer therapy, bone regeneration, neurogenesis, and implants [125]. Due to the existed negative PO_4_^3−^ on BP nanomaterials’ surface, positively charged chemotherapeutic or immunostimulatory agents can be directly loaded onto the surface of nano-scaled BP via electrostatic adsorption for synergistic cancer treatment. Meanwhile, neutral or negatively charged agents can also be loaded on BP nanomaterials after the modification of the payload or nano-scaled BP [126].

**Figure 5:**
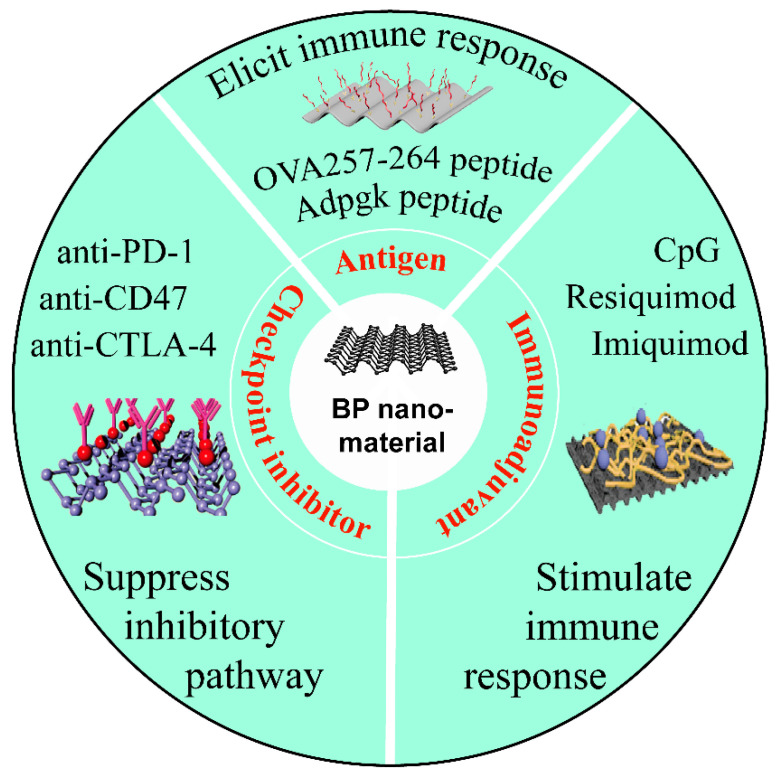
Schematic illustration of the delivery of immunotherapeutic agents through BP nanomaterials in cancer immunotherapy. The checkpoint inhibitor, antigen, and immunoadjuvant are three main types of immunotherapeutic agents which can be loaded on BP nanomaterials to elicit distinct effects for cancer immunotherapy.

Immunotherapeutic agents, including checkpoint inhibitors, vaccines, cytokines, and adjuvants, are commonly applied during clinical cancer immunotherapy. However, systemic delivery of immunostimulatory agents may cause potential side effects such as high immune-mediated toxicity, lack of targeted delivery, and massive degradation [127]. As is depicted in Figure 5, BP nanomaterials can be harnessed as smart nanocarriers for loading various immunotherapeutic agents to overcome these shortcomings. By virtue of targeting ligand and stimulus-triggering moieties under NIR irradiation, the constructed nanocomplexes combined immunotherapeutic agents with BP hold numerous advantageous properties, such as on-demand release, targeting delivery, enhanced stability, and improved immunotherapeutic performance.

### 5.1. Checkpoint Inhibitors Delivery

Immune checkpoint inhibitors (ICIs) are monoclonal antibodies that can suppress the inhibitory pathway such as cytotoxic T lymphocyte-associated protein 4 (CTLA-4) and the PD-1/PD-L1 axis [128]. ICIs can reverse immune suppression by binding to either the immune checkpoint molecule such as cytotoxic CTLA-4 and PD-1 or the immune checkpoint ligand such as PD-L1 [129]. Nivolumab is a type of PD-1/PD-L1 inhibitor that have been approved by FDA for the treatment of metastatic melanoma [130]. Through the plug-and-play nanorization system, Ou and colleagues prepared uniform BPNSs to load chemotherapeutics DOX (D) [131]. Subsequently, layers of positively charged chitosan-PEG (c) conjugated with folic acid (F) were covered onto BPNSs’ surface, which could not only be regarded as the targeting agent, but also conjugate negative cancer growth inhibitor siRNA (s) and programmed death ligand 1 (PL) for genetic intervention of the programmed death 1 (P)/programmed death ligand 1 (P/PL) pathway. With the presence of 808 nm laser, BP-DcF@sPL nanocomposites activated a higher proportion of CD11c^+^ CD86^+^ matured dendritic cells (DCs) (∼20%) and T cells (∼37%) in vivo than the control group in mice, which could further increase the expression levels of IFN-γ and enhance the anticancer therapy effect.

### 5.2. Antigen Delivery

Antigen delivery has emerged as a favorable approach for cancer immunotherapy, which is characterized with relatively low cost and high specificity to attack tumor cells with low side effects [132]. An effective cancer nanovaccine is often composed of tumor antigens and immunoadjuvants, which can elicit antigen-specific T-cell responses against tumor cells [133]. OVA257-264 peptide (SIINFEKL, OVAp), which belongs to the CD8^+^ T cell epitope, requires a carrier to antigen presenting cells [134]. Previous studies reported that bare BP was able to elicit immune responses with improved secretion of inflammatory [118]. Thus, BP can be harnessed as an adjuvant for antigen delivery. Here, OVAp and phenylalanine-lysine-phenylalanine (FKF) were integrated onto the surface of BP nanomaterials to obtain nanovaccines termed as FKF-OVAp@BP [135]. Within tumor mild acidic environment, the electrostatic interaction between lysine and BP nanomaterials was disturbed, subsequently inducing the dissociation of antigen FKF peptide from BP nanomaterials, which enabled OVAp-mediated antigen-specific immune responses regardless of in vitro or in vivo administration. Besides, the photothermal effects triggered by BP nanomaterials also facilitated local immune activation.

### 5.3. Immunoadjuvant Delivery

Generally, insufficient immunogenicity is a severe problem for vaccine application, leading to the failure of stimulating effective anticancer immune responses [136]. Therefore, immunostimulatory adjuvants are developed to both activate the innate immune responses and trigger tumor-specific immune responses [137]. Zhao and his colleagues reported the integration of bPEI-PEG coated BPNSs and CpG via electrostatic interaction [138]. Taking advantage of the necroptotic pathway mediated by BP nanomaterials based-PTT, damage-associated molecular patterns (DAMPs) were released from the necroptotic cells to reinforce the anticancer immune responses. In vivo experiments demonstrated that BP nanocomposite-based cancer therapy displayed much higher immunopotentiation than other control groups on bilateral 4T1 breast tumor model, where the percentage of antigen-specific CD8^+^ T lymphocytes and CD4^+^ T lymphocytes were 2.98% and 3.16% in tumor tissues, respectively. Notably, the anticancer cytokines IL-2, TNF-α and IFN-γ in the serum were significantly improved, which elicited enhanced immune response.

## 6. Synergistic Cancer Photoimmunotherapy Based on Black Phosphorus Nanomaterials

**Figure 6:**
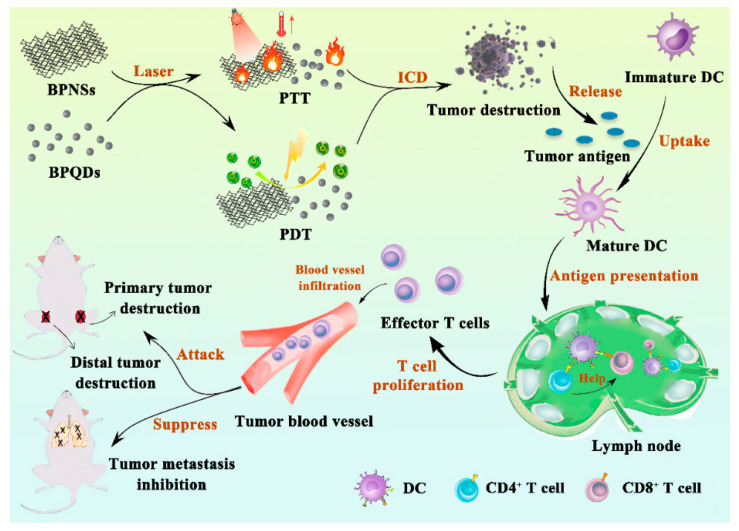
Schematic illustration of the synergistic cancer photoimmunotherapy based on BP nanomaterials. When exposed to specific laser irradiation during anticancer treatment, BP nanomaterials exhibit excellent phototherapy effect, which can not only destroy the tumor in situ, but activate tumor immunity as well. As a result, both subsequent tumor destruction and tumor metastasis inhibition can be acquired through this synergistic anticancer therapy.

BP nanomaterials are being widely investigated as an alternative besides conventional nanoplatforms [126]. Due to its great optical properties, BP nanomaterials have shown promising PTT and PDT effects when exposed to specific laser irradiation during cancer treatment [139]. Furthermore, PTT can also trigger the in situ release of tumor neoantigen, which is an effective strategy for personalized tumor immunotherapy (IT) [140]. As is described in Table 4 and Figure 6, a robust anticancer effect can be achieved when BP nanomaterials were adopted for loading and administrating immunoregulatory agents together for the combination of PTT/IT and PDT/IT. The combined cancer treatment modality can both improve their therapeutic efficacy and lower side effects.

### 6.1. Synergistic Photothermal-Immuno Therapy

PTT is an effective method for cancer therapy with minimal invasiveness and excellent selectivity [148]. Notably, BP nanomaterials-mediated photothermal ablation can lead to both localized hyperthermia for effective tumor cell eradication and the release of immunotherapeutics. For example, Shou and his coworkers designed a type of temperature-sensitive BPQDs hydrogel to encapsulate bisphosphonate drug zoledronate, an agonist for the expansion of γδ T cells [143]. Furthermore, zoledronate could be controllably released based on the photothermal effect of BPQDs. Compared with the free drug, the sustained release of zoledronate exhibited enhanced proliferation of γδ T cells, which secreted the cytotoxic factors granzyme B and perforin for robust anticancer activity against both in vitro breast and bladder tumor cells. In another study, Xie et al. employed the hyperthermic ablation of BP nanomaterials to improve the response rate of CD47 antibody (aCD47) [142]. After NIR irradiation, the PTT triggered by BPNSs not only mediated tumoricidal effect directly through photothermal ablation, but activated both innate and adaptive anticancer immune response through the dramatically increased monocytes and cytotoxic T lymphocytes (CTLs) in vivo as well. The administration of aCD47 could induce the repolarization of tumor-associated macrophages (TAMs) to M1-like macrophages, thereby enhancing the systemic anticancer immune response for destroying metastatic cancer. Taking advantage of the necroptotic pathway mediated by BP nanomaterials based-PTT, DAMPs were released from the necroptotic cells to reinforce the anticancer immune responses. In vivo experiments showed that the anticancer cytokines IL-2 and IFN-γ were significantly improved, which elicited enhanced immune response.

Interestingly, after the PTT treatment by BP nanomaterials, the released neoantigen can activate specific effector T cells for the further elimination of tumor cells [133]. Liang et al. constructed erythrocyte membranes (RMs)-coated BP quantum dot biomimetic nanovesicles (BPQD-RMNVs) [93]. BPQDs exhibited excellent NIR photothermal performance to cause apoptosis and necrosis of basal-like 4T1 breast tumor, which led to the recruitment of DCs and further captured neoantigens in vivo. Subsequently, the administrated aPD-1 increased the infiltration and activity of CD8^+^ T cells, thereby stimulating the immune system and delayed primary and distal tumor growth.

As the membrane composition of exosomes is similar to its parent cell with different biological functions, the specific origin is carefully selected when utilizing exosomes for targeted drug delivery [149]. Serum exosomes (hEX) derived from hyperthermia-treated tumor-bearing mice contain relevant tumor-associated antigens (TAAs), involving the differentiation and maturation of immunoregulatory-like DCs [150,151]. Thus, Das et al. employed hEX as adjuvant-like therapeutic nanocarriers to encapsulate BPQDs [141]. The developed tumor vaccine (hEX@BPNSs) exhibited an enhanced Th-1 immune response, and higher levels of IL-2, IL-6, IFN-γ, and TNF-α were detected in vivo. Furthermore, PTT can increase the tumor infiltration of immunocytes with a significantly higher percentage of CD4^+^ T and CD8^+^ T cells. Due to the synergistic effects of PTT and immunotherapy, the mouse lung cancer model group treated with hEX@BP plus NIR exhibited better anticancer effects with delayed tumor growth and prolonged survival time than other groups.

In addition, local hyperthermia can promote local circulation and vascular permeability to facilitate the tumor penetration of T cells, which helps to improve anticancer immune responses and inhibit tumor growth [152]. Therefore, a more outstanding anticancer effect can be achieved through the synergistic PTT/IT based on BP nanomaterials.

### 6.2. Synergistic Photodynamic-Immuno Therapy

PDT is a technique with high selectivity and low systematic toxicity for cancer treatment [153]. It has been demonstrated that BP nanomaterials can be applied as PS under suitable laser irradiation to generate cytotoxic ROS [139]. Guo et al. have verified that PEG stabilized BPQDs could effectively generate ROS to kill cancer cells under light irradiation through both in vitro and in vivo studies [59]. Upon NIR laser irradiation, the triggered ROS from BP nanomaterials not only induces tumor cell apoptosis, but can be used to achieve ROS-responsive release of loaded therapeutic agents as well.23

Argentum ions (Ag^+^) can be captured by macrophages to stimulate proinflammatory responses. Thus, Ag^+^-based nanocomplexes can exhibit outstanding application prospect in immune-related therapy. Li et al. utilized ROS-sensitive polypropylene sulfide (PPS) to construct an NIR light stimulus responsive BPQDs vesicle (BPQD NVs) for delivering Ag^+^ [145]. Under NIR laser irradiation, the generated ROS by BPQD NVs-Ag^+^ could induce ICD and control the release of Ag^+^. In vitro results confirmed that BPQD NVs-Ag^+^ could induce a strong ICD effect with the most considerable exposed calreticulin (CRT). Moreover, BPQD NVs-Ag^+^ with NIR treated cancer cells expressed the highest level of proinflammatory cytokines to promote the ICD effect by BPQDs. With the co-stimulation of Ag^+^ and local PDT, the mice treated with BPQD NVs-Ag^+^ significantly enhanced DCs’ maturation. Furthermore, the infiltration of CD8^+^ T cells and CD4^+^ T cells were remarkably promoted in the distal tumor. Therefore, the occurrence of tumor nodules was suppressed to 10.45%. This study indicated that the synergistic photodynamic/Ag^+^ therapy for tumor treatment is capable of eliciting a potent immunotherapy response, thereby inhibiting the distant tumor growth and lung metastasis of the breast tumor.

Besides, Li and his teammates prepared another amphiphilic NIR/ROS sensitive BPQD nanovesicles (BPQD NVs) loaded with oligodeoxynucleotides (ODNs) containing CpG motifs (CpG-ODNs), which were formed by the self-assembly of PEG and ROS sensitive PPS grafted BPQDs [146]. Apart from the direct tumoricidal effect, the generated ROS from BPQD NVs-CpG also triggered the disintegration of PPS, which further caused the decomposition of the vesicles to release free BPQDs and CpG at tumor site. The on-demand released CpG could enhance the secretion of pro-inflammatory cytokines to induce an improved anticancer immune response through the activation of cytotoxic T cells. Among all treatments, the 4T1-tumor-bearing mice treated with BPQD NVs-CpG and laser irradiation exhibited the best tumor suppression effect and the highest survival rate. Moreover, lung metastasis and distant tumor growth were also effectively prevented, indicating the outstanding synergistic PDT/IT effect based on the stimuli-responsive BPQD NVs-CpG nanoplatform.

### 6.3. Synergistic Photothermal-Photodynamic-Immuno Therapy

The application of a positive feedback strategy is a novel idea that can gradually improve the therapeutic effect during anticancer treatment. Su et al. synthesized a TGF-β inhibitor LY364947 (LY)-loaded BPNSs with the modification of both PEI and neutrophil membrane-derived nanoghost (NG), obtained the nano DDS named NG/BP-PEI-LY [147]. Acute local inflammation could be induced through PTT and PDT, while NG possessed high affinity to the inflammatory site [154,155]. Therefore, more NG modified NPs could precisely target to the tumor site after PTT and PDT. Furthermore, the increased amount of accumulated NPs in the tumor in situ further enhanced the local photothermal and photodynamic effect, ensuring more NPs target to tumor site, eventually producing the positive feedback targeting effect. Moreover, this synergistic anticancer therapy based on PTT and PDT combined with LY effectively increased the infiltrating amount of CD4^+^ T cells and CD8^+^ T cells, while the number of T regulatory cells (Tregs) in the tumor significantly decreased after the aforementioned anticancer therapy. In addition, this tumor immunotherapy effect was gradually enhanced due to the mechanism of positive feedback targeting. Thus, both the growth of the in situ tumor and the tumor metastasis were effectively inhibited through this combinational anticancer therapy.

## 7. Summary and Outlook

This review gives a detailed introduction of BP nanomaterials and summarizes the recent advance of its application in cancer therapy, especially from the aspect of immunotherapy. BP is a novel inorganic material with bright application prospects in various fields. Due to its broad absorption band of the visible spectrum and unique electronic structure, BP possesses high a photothermal conversion ability and can produce cytotoxic ROS, enabling effective tumor cell death. BP nanomaterials, which can be further classified into BPNSs and BPQDs, are a type of excellent nanoplatform in anticancer drug delivery. BP nanomaterials possess outstanding biocompatibility and biodegradability in vivo, which facilitates their application in the biomedical field. However, the instability of BP in ambient conditions is a bottleneck that limits its effectiveness. Therefore, physical or chemical surface modification are adopted to enhance the stability of this nanomaterial. In addition, surface modification also endows BP nanomaterials with special properties which are closely relevant to the unique properties of distinct modifiers, further ensuring the synergistic therapeutic efficacy after administration.

The application of BP as nanocarriers to deliver chemotherapeutics and immunostimulatory agents due to its superior drug loading capability can protect the corresponding therapeutic agents from degradation or inactivation as well as reduce their dosage with decreased side effects. In addition, upon laser irradiation, nano DDS, based on BP nanoplatforms, performs an excellent PTT and PDT effect, directly leading to the tumor killing effect during cancer treatment. Therefore, BP-mediated phototherapy provides the first line of defense against the tumor, which is able to effectively eliminate the primary tumor. Subsequently, the released TAAs and DAMPs through the induction of ICD in target tumors can trigger an immune response and strengthen the immunotherapy effect. Besides, the pro-inflammatory cytokines, which can activate the immune system, are also elevated. In a nutshell, the PIT effect generated through BP is capable of circumventing the challenges of tumor heterogeneity, tumor mutation, and tumor immune escape, as well as increasing immunogenicity of the tumor microenvironment, eventually recruiting more antigen-presenting cells (APCs) or decreasing immunoregulatory suppression. Hence, the application of BP in cancer PIT opens a new era for the current anticancer therapy and also provides more opportunities for patients with metastatic tumors.

Despite the merits mentioned above, there are still many obstacles that are needed to be overcome before clinical transformation of BP nanomaterials. For example, the preparation cost of both bulk BP and BP nanomaterials is relatively high, which may obviously increase the treatment cost to patients if applying BP nanomaterials in cancer therapy. In addition, due to the complicated preparation process, the controllable industrial production of BP nanomaterials is another tricky problem, which restricts their successful clinical application. Besides, it is also difficult to reproduce products with the same quality at large-scale preparation. Moreover, the clinical application of PTT and PDT for tumor treatment is still immature to some extent. The boundedness of phototherapy is its limited tissue penetration of light, which restricts the non-invasive application of phototherapy to treat tumors in deep organs. Therefore, phototherapy is more suitable for superficial cancers, such as melanoma, osteosarcoma, and squamous cell carcinoma.

Nevertheless, with the rapid development of modern technology and expanded preclinical and clinical studies, we are convinced that PIT mediated by BP nanomaterials will have a bright future and the obstacles that hinder their clinical applications will be overcome one day. BP is still a promising nanomaterial with great research potential and clinical interest.

## Figures and Tables

**Table 1:** Examples of various synthesis routes for the preparation of BP nanomaterials.

Classification of BP Nanomaterials	Synthesis Routes	Properties of the Prepared BP Nanomaterials	Ref.
BPNSs	Tape exfoliation method	The thickness is about 3 nm, and the lateral size is more than 50 μm	[46]
	Wet ball milling exfoliation method	The lateral size is between 1 and 5 μm, and the flakes are generally 3–5 layers	[47]
	Ultrasonic-assisted liquid-phase exfoliation method	The thickness ranges from 2.06 to 9.4 nm with single or few layers	[33,48,49]
	Electrochemical exfoliation method	The lateral size ranges from 0.5 to 30 μm, and the thickness is from 1.4 to 10 nm with single or few layers	[50,51,52]
	Chemical vapor deposition method	Commonly with 4 layers’ nanoflakes	[53]
	Plus-laser deposition method	With the thickness from 2 to 8 nm	[54]
	Solvothermal method	The lateral size is between 0.8 and 2.0 μm, and the thickness is of about 4.7 nm with approximately 9 layers’ flakes	[55]
	Hydrothermal method	The lateral size is of approximately 5 μm, and the thickness is of around 3 nm with 1–2 layers’ flakes	[56]
BPQDs	Ultrasonic exfoliation method	The average size is about 2.5 nm, and the average height is around 1.4 nm	[31,57]
	Solvothermal method	The average size is about 2.1 nm, and the average height is around 3 nm	[58,59]
	Pulsed laser ablation method	The average size is about 6 nm, and the approximate average height is 1.1 nm	[60]
	Blender breaking method	The average size is about 2.2 nm, and the average height is between 0.58 and 1.45 nm	[61]

**Table 2:** Examples of various physical modification methods to BP nanomaterials.

Modification Methods	Modifiers	Nanoagents	Modification Effects	Ref.
Modification Using Polymer	PLGA	BPNSs/PLGA/DOX	Ensure BPNSs’ PTT effect and biocompatibility, produced combined therapeutic effect	[78]
		BPQDs/PLGA	Obtain controllable degradation rate of BPQDs, ensure BPQDs’ photothermal stability, biocompatibility, and long circulation in vivo	[38]
		BPQDs/DTX@PLGA	Enhance BPQDs’ biocompatibility, promote chemophotothermal combinatory therapeutic efficacy, and tumor targeting effect through EPR mechanism	[79]
		MSC@BPQDs/PLGA	Improve the uptake of the nanoagent by MSCs, enhance the nanoagent’s stability, and exhibit tumor specific tropism	[80]
		PLGA_-ss-_D@BPQDs	Enhance radiotherapy efficacy, improve accurate tumor tissue localization through RGD targeting, surface charge switching, and bioresponsiveness, and reduce toxicity	[81]
	PEG	BP-PEG NSs + R837	Remarkably enhance photothermal stability, elicit a strong immune response through both PTT and R837	[82]
		BP@PEG/Ce6 NSs	Improve biocompatibility, physiological stability, tumor-targeting property, and photothermal conversion efficiency (43.6%)	[24]
		RdB/PEG-BPQDs	Enhance biocompatibility, physiological stability, and bioimaging property	[31]
		DF-PEG-PAHy/BPNSs	Exhibit excellent gelation characteristics, pH sensitivity, NIR responsiveness, good biocompatibility, and outstanding photothermal characteristics	[83]
	PDA	BP@PDA-Ce6 and TPP NSs	Improve the photothermal conversion efficiency and the stability of BPNSs, provide amine anchors for further functionalization by Ce6	[84]
		BPQDs@PDA	Efficiently prevent the oxidation of BPQDs due to the enriched phenol groups on PDA, improve the photothermal conversion efficiency	[75]
		BPNSs-DOX@PDA-PEG-FA	Enhance stability, photothermal efficiency, and targeting ability for cancer cells	[85]
		BPNSs-DOX@PDA-PEOz-BTZ	Improve targeted long circulation and cellular uptake in vivo	[34]
	Polypeptide	BPNSs@FKK	Exhibit excellent stability, favorable cell compatibility, enhance cellular uptake, and increase life span of the nanoagent	[86]
	CS	CS@BPNSs@CuNPs	Possess a remarkable temperature-sensitive spongy-like state, increase ROS production, improve postoperative therapy and multi-tumor treatments	[87]
	PLL	PLL/BPNSs/Cas13a/crRNA	Enhance cell adhesion and membrane penetration, improve stability in physiological solutions, enable the load of Cas13a/crRNA complexes	[88]
	PEI	BPNSs-PEI/AuNPs	Serve as ’bridge‘ to form BPNSs-PEI/AuNPs, increase ^1^O_2_ production, and enhanced light absorption	[89]
Modification Using Inorganic Nanomaterials	Metallic Modification	BP-Au NSs	Enhance photothermal efficiency, serve as SERS substrates for Raman biodetection	[90]
	Nonmetallic Modification	BPNSs@MS/PEG/TKD peptide/DOX	Enhance BPNSs’ dispersity, drug-loading efficiency, and post-modification feasibility	[91]
		RGD-Ir@BPNSs	Improve photoelectric properties, photo-induced charge carrier dynamics of BPNSs, and singlet oxygen generation after X-ray irradiation	[92]
Modification Using Lipid Based Nanocarrier	Cell Membrane	BPQD-RMNVs + aPD-1	Improve long circulation time and tumor accumulation in vivo, prevent CD8^+^ T cells from exhaustion	[93]
		Gel-BPQD-CCNVs+ aPD-1	Serve as tumor vaccine, exhibit strong and durable PTT effect and immunological response, inhibit tumor recurrence and metastasis	[94]
	PLT Membrane	PLTm@BPQDs-HED	Enhance tumor targeting ability, mitochondria-mediated cell apoptosis and autophagy in tumor cells, prolong in vivo circulation time	[95]
	Liposome	RV/CAT-BPNSs@MFL	Possess excellent stability, good photo-controlled release behavior of drug, high photothermal conversion efficiency, enhance singlet oxygen release efficiency, and outstanding tumor-targeting ability	[96]
		Adpgk-BPQDs-liposome@F127 gel	Enable the co-encapsulation of colon cancer cells derived neoantigen peptide Adpgk with BPQDs, enhance immunostimulatory effect, drug stability, and in vivo circulation time	[97]

**Table 3:** Examples of various chemical modification methods of BP nanomaterials.

Modification Methods	Modifiers	Nanoagents	Modification Effects	Ref.
Modification Using Small Molecule	NB	NB@BPNSs	Exhibit improved PTT and NIR imaging efficiency, possess good biocompatibility	[117]
	TIL_4_	TiL_4_@BPNSs	Improve biocompatibility, reduce cytotoxicity, proinflammation, and adverse immune responses, circumvent macrophage’s uptake	[118]
Modification Using Polymer	TPP	BPQDs-PEG-TPP	Exhibit excellent mitochondria-targeted property, NIR photothermal properties, ROS production ability, stability, and dispersibility	[119]
Modification Using Metallic nanomaterial	PtNPs	BP/Pt-Ce6@PEG NSs	Ameliorate hypoxia and photodynamic effect, Improve the modifiability of nano DDS	[120]

**Table 4:** The summarized applications of BP nanomaterials for synergistic PIT.

Treatment Modalities	Nanoagents	Anticancer Efficacies	Ref.
PTT/IT	BPNSs-DcF@sPL	Circumvent PL pathway-regulated immune tolerance and suppression of CD8^+^ T cells, enhance the IFN-γ expression and promote the survival rate	[131]
	FKF-OVAp@BPNSs	Enhance antigen uptake, activate systemic immunity and prolong the survival time	[135]
	BPNSs-bPEI-PEG-CpG	Induce necroptotic cell death, activate T lymphocytes, increase serum IL-2, TNF-α and IFN-γ level, suppress both primary and distal tumors	[138]
	hEX@BPNSs	Activate immune system, inhibit tumor progression and prolong survival	[141]
	BPNSs + aCD47	Enhance the infiltration amount of CD8^+^ and CD4^+^ T cells in the tumor, increase the secretion of IL-6 and IFN-γ in the serum, suppress the tumor progression of both local and distal tumors	[142]
	BPNSs-PEG + R837	Stimulate pro-inflammatory cytokine release, increase the percentage of CD8^+^ T cells in the spleen, inhibit tumor growth	[82]
	BPQD-RMNVs + aPD-1	Increase the infiltration and activity of CD8^+^ T cells in the tumor, improve the serum IFN-γ levels, inhibit primary and secondary tumor growth	[93]
	Gel-BPQDs-CCNVs + aPD-1	Upregulate the expression of Ki67 in the main immune cells, increase the level of IFN-γ and TNF-α, avoid cancer recurrence and metastasis	[94]
	Adpgk-BPQDs-liposome@F127 gel	Induce OVAp-specific splenocyte proliferation, increase the level of active CD8^+^ T cells in the spleen, prevent the tumor progress	[97]
	BPQDs@pNIPAM-zoledronate	Control the release of drug, promote γδ T cell proliferation and inhibit tumor growth	[143]
	FePt/BPNS-PEI-FA + CTLA-4 blockade	Induce DC maturation, upregulate DC-secreted immune-related cytokines (TNF-α, IL-12, IFN-γ), control the growth of residual and metastatic tumor	[144]
PDT/IT	BPQD NVs-Ag^+^	Upregulate the secretion of inflammatory-related cytokines (TLR4 and IL-1β) in the tumor microenvironment, activate anticancer IFN-γ^+^ CD8^+^ T cells to distal tumor and provoke T cell-mediated immunity, suppress tumor growth and metastasis	[145]
	BPQD NVs-CpG	Increase serum levels of TNF-α, IL-6, and IL-12, enhance the tumor-infiltrating CD8^+^ T cells and the proliferation of T cells in the distal tumor, inhibit tumor growth and lung metastasis	[146]
PTT/PDT/IT	NG/BPNSs-PEI-LY NPs	Produce acute inflammation in tumor in situ after PTT and PDT, improve the accumulation of NPs by NE membrane-mediated affinity based on positive feedback strategy, induce potent immune activation in tumor in situ and effectively inhibit lung metastasis through PTT and PDT combined with TGF-β inhibitor	[147]

## Abbreviations

aCD47	CD47 antibody
Adpgk	Adpgk neoantigen peptide
Ag^+^	Argentum ion
aPD-1	Programmed cell death protein 1 (PD-1) antibody
Au	Aurum
CCNV	Cancer cell membrane nanovesicle
CS	Chitosan
CTLA-4	Cytotoxic T lymphocyte-associated protein 4
FKF	Phenylalanine-lysine-phenylalanine
FKK	Fmoc-Lys-Lys-Phe peptide
hEX	Serum exosomes
Ir	Iridium
MFL	Multifunctional liposome
MS	Mesoporous silica
MSC	Mesenchymal stem cell
NB	Nile Blue
NG	Neutrophil membrane-derived nanoghost
NIPAM	N-isopropylacrylamide
NVs	Nanovesicles
OVAp	OVA257-264 peptide
PDA	Polydopamine
PEG	Polyethylene glycol
PEI	Polyetherimide
PL	Programmed death ligand 1
PLGA	Poly (lactic-co-glycolic acid)
PLL	Polylysine
PLT	Platelet
PLTm	Platelet membrane
PtNPs	Platinum nanoparticles
R837	Imiquimod
RdB	Rhodamine B
RMNV	Erythrocyte membrane nanovesicle
TIL_4_	Titanium sulfonate ligand
TPP	Triphenylphosphine
